# Raman Spectroscopy Characterization of Multi-Functionalized Liposomes as Drug-Delivery Systems for Neurological Disorders

**DOI:** 10.3390/nano13040699

**Published:** 2023-02-11

**Authors:** Francesca Rodà, Silvia Picciolini, Valentina Mangolini, Alice Gualerzi, Pierfausto Seneci, Antonio Renda, Silvia Sesana, Francesca Re, Marzia Bedoni

**Affiliations:** 1 IRCCS Fondazione Don Carlo Gnocchi ONLUS, 20148 Milan, Italy; 2 Clinical and Experimental Medicine PhD Program, University of Modena and Reggio Emilia, 41121 Modena, Italy; 3 Department of Molecular and Translational Medicine, University of Brescia, 25121 Brescia, Italy; 4 Chemistry Department, Università Degli Studi di Milano, 20133 Milan, Italy; 5 School of Medicine and Surgery, University of Milano-Bicocca, 20854 Vedano al Lambro, Italy

**Keywords:** liposome, Raman spectroscopy, drug delivery, neurological disorders, therapeutics, multivariate analysis

## Abstract

The characterization of nanoparticle-based drug-delivery systems represents a crucial step in achieving a comprehensive overview of their physical, chemical, and biological features and evaluating their efficacy and safety in biological systems. We propose Raman Spectroscopy (RS) for the characterization of liposomes (LPs) to be tested for the control of neuroinflammation and microglial dysfunctions in Glioblastoma multiforme and Alzheimer’s disease. Drug-loaded LPs were functionalized to cross the blood–brain barrier and to guarantee localized and controlled drug release. The Raman spectra of each LP component were used to evaluate their contribution in the LP Raman fingerprint. Raman data analysis made it possible to statistically discriminate LPs with different functionalization patterns, showing that each molecular component has an influence in the Raman spectrum of the final LP formulation. Moreover, CLS analysis on Raman data revealed a good level of synthetic reproducibility of the formulations and confirmed their stability within one month from their synthesis, demonstrating the ability of the technique to evaluate the efficacy of LP synthesis using small amount of sample. RS represents a valuable tool for a fast, sensitive and label free biochemical characterization of LPs that could be used for quality control of nanoparticle-based therapeutics.

## 1. Introduction

Liposomes (LPs) are phospholipid-bilayered nanoparticles enclosing an aqueous core that have been widely investigated for the development of pharmaceutical formulations as drug delivery platforms. Thanks to their versatile composition and structure, LPs can hold both hydrophilic and lipophilic drugs and can be functionalized with several molecules according to the expected application. LP properties can be modulated through design and synthesis to provide a controlled release of drug cargo and to target specific regions, both important features to obtain optimized drug-delivery systems [1].

A complete characterization of a LP-based nanoformulation is necessary for quality control, in order to predict and guarantee efficiency and safety in biological systems. However, it often represents a complex step in batch preparation due to the variety of the physicochemical properties to be measured. In particular, particle size distribution, surface charge, morphology and encapsulation efficiency need to be defined and optimized in order to obtain reproducible product batches to be successfully developed as nanocarriers [2].

Several analytical strategies and technologies can be applied for the characterization of liposomal pharmaceutical formulations, from chromatography approaches to light-based methods, from colorimetric and fluorimetric assays to microscope techniques. Dynamic light scattering (DLS) is the most common technique for determining particle size and polydispersity index (PDI) [3]. Morphology is frequently assessed using high-resolution microscopy techniques such as transmission electron microscopy (TEM) and cryo-TEM [4] or atomic force microscopy, which enable visualization of single particles, evaluating the liposomal shape, which could be transformed by the encapsulation of specific drugs or surface functionalization [5,6]. Additionally, several methodologies are available to determine the amount of molecules encapsulated within LPs, and include high-performance liquid chromatography, ultraviolet-visible spectrophotometry and capillary electrophoresis. For lipid quantification, spectrophotometric assays or chromatographic approaches are usually employed. The physical and chemical stability of LPs are assessed by using spectroscopy strategies, DLS and chromatography approaches to measure liposome fusion, aggregation, and degradation [7,8].

Among reported techniques, Raman spectroscopy (RS) is a vibrational spectroscopy that provides a specific spectrum for each analyzed sample, called a fingerprint. Indeed, the positions and the intensities of the peaks are related to the biochemical composition of the specimen. RS is suitable for the characterization of LPs in order to test their reproducibility, the effective functionalization with selected ligands, and their stability using a small sample amount, rapidly obtaining results. Raman analysis of LPs is not common, but has been used to directly visualize the distribution of sphingomyelin tagged with a Raman active molecule in lipid rafts of monolayer membranes, as well as the discrimination between multiple components of lipid membranes in a label-free manner, paving the way for a quantitative analysis of lipids [9]. Another study showed that RS may determine the distribution of active pharmaceutical ingredients in several formulations, including LPs, being indicative of the cellular uptake or penetration through membranes [10]. RS was also used to determine the biochemical composition of cationic liposomal drug-delivery systems in vitro, and to evaluate the extent of mitochondrial association, depending on the liposome composition [11]. More recently, RS analysis, supported by X-ray diffraction patterns and DSC thermograms, was applied to the identification and physicochemical characterization of a poorly bioavailable molecule encapsulated in LPs to overcome solubility and stability problems limiting its medical application [12]. 

In the present work, RS was applied for the quality control of several batches of multi-functionalized LPs designed as putative therapeutics for the control of neuroinflammation and associated microglial dysfunctions in Glioblastoma multiforme (GBM) and Alzheimer’s disease (AD), two neurological diseases that dramatically impact the worldwide health and economy. Although GBM has a low incidence (<1%) compared with other tumors, its high lethality results in a survival rate of less than 2 years [13]. Instead, about 50 million people are affected by AD, and the number is increasing rapidly due to the population aging and living longer [14]. Additionally, drug delivery to the brain is still challenging because of the presence of the blood–brain barrier (BBB), which limits drugs’ access to the brain. In this framework, LPs have been tested to evaluate their therapeutic potential as drug-delivery systems that can improve the brain bioavailability of glibenclamide, pimasertib and trametinib. Glibenclamide has been selected as AD-targeted drug for its ability to inhibit the inflammasome-mediated production of IL-1 β by blocking P2X7 ion channels involved in microglia activation [15]. Pimasertib and trametinib have been identified for GBM treatment. The former is a highly selective allosteric inhibitor of Mek1/2 that has entered clinical trials for several solid tumors [16,17,18], while trametinib is the first FDA-approved MEK1/2 inhibitor currently in use for unresectable and metastatic melanoma. Both anticancer molecules target the mitogen-activated protein kinase pathway, which is hyper-activated in most of GBM and requires signaling via MEK kinase to increase cellular proliferation and survival [19]. Despite their high potential, the three free molecules have failed to achieve therapeutic levels in the brain due to poor bioavailability [20]. To overcome this problem, we developed properly functionalized liposome-based formulations as drug-delivery systems. The proposed LPs were loaded with the selected drugs and functionalized with a peptide derived from apolipoprotein E (mApoE) to enhance BBB crossing, and with a lipopeptide, called SG-17, for targeting specific matrix metallo-proteases (MMPs) overexpressed in the neuroinflammatory and tumor niche. The ability of mApoE to increase the BBB penetration of LPs has already been demonstrated in in vitro and in vivo models [21,22,23], while surface modification with SG-17 has been shown to determine liposomal destabilization and cargo release [24]. Moreover, the ability to cross the BBB of LPs functionalized with both mApoE and SG-17 has already been demonstrated in vitro [25]. As the chemical characterization of LPs is a crucial step for their development and validation in a clinical setting, we tested RS as a sensitive, fast and cheap method to evaluate and optimize the efficacy of LP synthesis, by collecting the specific Raman fingerprint of each formulation.

Our results demonstrated the ability of this biophotonics-based tool to obtain a specific chemical fingerprint for LP formulations and to identify the contribution of each component in the LPs Raman spectra using a small sample volume, showing the power of RS for the quality control of LP synthesis, the evaluation of reproducibility between different batches and the stability assessment of LP formulations in time.

## 2. Materials and Methods

### 2.1. Lipopeptide Construct Synthesis

SG-17, an MMP-sensitive lipopeptide (SKK(stearate)SGPLGIAGQSK(stearate)KS), was prepared by microwave (MW)-assisted Fmoc solid-phase peptide synthesis (Fmoc-SPPS) on Wang resin (loading between 0.2–0.4 mmol/g), using a CEM Liberty Blue synthesizer. Coupling reactions were performed with amino acids (5 eqs., 0.2 M in DMF), using diisopropyl carbodiimide (DIC, 0.5 M in DMF) and Oxyma Pure (1 M in DMF) as coupling reagents. The MW synthesis cycle entailed 15 s at 75 °C–170 W, followed by 110 s at 90 °C–40 W. N-Fmoc deprotection was performed using 20% piperidine in DMF with a MW cycle entailing 15 s at 75 °C–155 W, followed by 60 s at 90 °C–50 W. Final cleavage in solution entailed shaking the resin for 3 h in a 90:2.5:2.5:5 TFA/TIPS/H_2_O/phenol mixture. The lipopeptide was then purified in reverse-phase chromatography with a Biotage Isolera instrument equipped with a C18 column. 0.1% TFA in water was used as phase A and 1% TFA in ACN as phase B, going from 20 to 100% phase B in 18 min (Rt = 16.6 min for MSLP-1). Electrospray ionization high-resolution time-of-flight mass spectrometry (ESI-HR TOF-MS) on a Q-TOF Synapt G2-Si yielded m/z 1503.7206 [M+2H]^2+^, 702.8170 [M+3H]^3+^ values for the pure lipopeptide. 

### 2.2. Liposomes Synthesis and Functionalization 

Conventional LPs were prepared as described previously [26]. Briefly, cholesterol (Chol; Merck KGaA, Darmstadt, Germany), sphingomyelin from bovine brain (Sm) and 1,2-Distearoyl-sn-glycero-3-phospho-ethanolamine-N[maleimide(polyethyleneglycol)-2000] (DSPE-PEG2000-mal; Avanti Polar Lipids, Inc, Alabaster, AL, USA), respectively, in a 48.75:48.75:2.5 molar ratio, were dissolved in chloroform in a round-bottom flask and mixed for 5 min. A phospholipid film was obtained through rotary evaporation, and then hydrated by addition of phosphate buffered saline (pH 7.4) for 1 h at 55 °C. After hydration, unilamellar LPs were obtained by probe sonication in a continuous mode for 20 min with 30% power delivery (probe type ultrasound Sonics & Materials, Inc., Newtown, Connecticut, CT, USA). To prepare SG-17 LPs, the lipopeptide was dissolved in methanol at 0.2 mg/mL, and then added to lipids previously dissolved in chloroform and mixed, with a 1:10 *w*/*w* SG-17:Sm ratio. Solvents were removed by rotary evaporation and LPs were prepared as described above. 

For targeting purposes, SG-17 LPs were surface-decorated by covalent coupling with mApoE peptide (CWGLRKLRKRLLR; Karebay Biochem, Monmouth Junction, NJ, USA) on DSPE-PEG2000-mal. A final 1:10 molar ratio (mApoE:DSPE-PEG2000-mal, mol:mol) was used. Size, PDI, and ζ-potential were determined by DLS and interferometric Doppler velocimetry (Brookhaven Instruments Corporation, Holtsville, NY, USA equipped with a ZetaPALS device). LPs (±SG-17 ± mApoE) were loaded with glibenclamide (G0639; Merck), trametinib (JTP-74057; CliniSciences, Nanterre, France) or pimasertib (AS-703026; CliniSciences). Each drug (0.5 mg), dissolved in an appropriate solvent, was added to a mixture of lipids (5 µmol of total lipids dissolved in 9 mL chloroform) and SG-17 (0.2 mg/mL, dissolved in methanol). Solvents were removed by rotary evaporation, LPs were prepared as described above, and purified and characterized as previously described [27]. Drug encapsulation yields were calculated by NanoDrop OneC Technology (Thermo Scientific, Waltham, Massachusetts, MA, USA), measuring the absorbance of the non-encapsulated drug compared to the drug amounts loaded into the LP batch using a free drug calibration curve. Lipid recovery was estimated by Stewart assay [28]. Encapsulation efficiency (EE%) and drug-to-lipid mass ratio (D/L, μg/μg) were calculated as described previously [29].

### 2.3. Raman Acquisition of Single Liposomal Components

Raman analyses were performed using a Raman microscope (LabRAM Aramis, Horiba Jobin Yvon S.A.S, Lille, France) equipped with a laser source operating at 532 nm. Single components of LPs were analyzed both as powders and resuspended in PBS, to mimic synthetic conditions. For each molecule, a few grains of powder and a 3 μL drop of LP component in PBS were laid separately on a CaF_2_ disk (Crystran LTD, Poole, UK). The silicon band at 520.7 cm^−1^ was used as reference for calibration. The square-map for acquisition (60 × 60 μm) was selected using a 50× objective (Olympus, Tokyo, Japan). Raman spectra of each sample were collected from 500 to 1800 cm^−1^ and from 2700 to 3200 cm^−1^, with a spectral resolution of 0.8 cm^−1^/step for the first range and 0.6 cm^−1^/step for the second. Raman spectra acquisition was performed using LabSpec6 software (version 6.4, Horiba Scientific SAS, Palaiseau, France). In order to create a Raman database of single components, acquisition parameters were adapted to each sample to obtain the most informative fingerprint (Table S1).

### 2.4. Raman Data Analysis

Raman spectra were analyzed using LabSpec6 (Horiba Scientific) and OriginLab2022 software packages. All the acquired spectra were fit with a polynomial baseline, resized on the reference band at 1438 cm^−1^ and 2847 cm^−1^, and normalized through unit vector normalization. Polynomial smoothing was also applied. The specific Raman fingerprint of LP components and of the different liposomal formulations was obtained by determining the average spectrum for each sample. 

Multivariate analysis was used to evaluate the ability of RS to distinguish different liposomal formulations. Principal Component Analysis (PCA) was performed for data dimensionality reduction, and the first two PCs were extrapolated and used for data representation. Then, Linear Discriminant Analysis (LDA) was applied to discriminate the Raman data and maximize the variance. One-way ANOVA or Mann–Whitney test was then used to verify any statistical differences among the groups. 

To determine the contribution of each component to the Raman spectrum of a complete LP formulation, the Classical Least Squares (CLS) fitting analysis method was applied using normalized average spectra of single components as reference and calculating the percentage of the scores from CLS for each component. 

### 2.5. Raman Acquisition of Liposome Formulations

Liposomal formulations were analyzed by RS using a 532 nm laser source. A drop of 3 µL of LPs was deposited on a CaF_2_ disk and dried before the acquisition. A 100× objective (Olympus, Japan) was used, with acquisition time of 25 s, 2 accumulations, grating at 1800 grooves/mm, slit at 200 µm and hole at 400 µm. For each formulation, 15–20 spectra were acquired at the border of the air-dried drop, in two spectral ranges—between 500 and 1800 cm^−1^ and between 2700 and 3200 cm^−1^. Table 1 summarizes the analyzed samples, including unloaded naked LPs made with Sm, Chol and DSPE-PEG-mal (LPs); unloaded LPs functionalized with mApoE (mApoE LPs) or with lipopeptide SG-17 (SG-17 LPs); and drug-loaded LPs functionalized with SG-17 and mApoE (SG-17 mApoE LPs + G/T/P). 

## 3. Results

### 3.1. LP Preparation

We synthetically targeted SG-17 (Figure S1), centered on an MMP2-responsive sequence (GPLGIAGQ)20 flanked on both sides by Ser and Lys as polar and protonated residues to increase its hydrophilicity, and connected with two hydrophobic tails; this hybrid peptide–lipopeptide sequence, once embedded into a hybrid liposome, provides sensitivity to MMP2 and selective cargo release in pathogenic microenvironments [25]. The seventeen-aminoacid-membered SG-17 lipopeptide was successfully prepared in good yields and purity (>90%) by microwave-assisted solid-phase peptide synthesis (MW-SPPS), using standard Fmoc-based automated protocols, as described in the Materials and Methods; N-Fmoc Ser, Lys, Ile, Ala, Gly, Pro and Leu were used as building blocks together with Nα-Fmoc-(Nε-stearoyl)-Lys, prepared according to [30].

LP characterization (Table S2) showed a uniform size distribution (PDI ≤ 0.2) with a <200 nm diameter and a negative net surface charge, suggesting that LP dispersions are stable and not prone to aggregation. A slight increase in LP size (ranging from 5–10%) was detected after surface functionalization with mApoE. These parameters are typical of homogenous samples with a stable profile. The values of drug EE% were 74 ± 11%, 92 ± 4% and 85 ± 4% for glibenclamide, trametinib and pimasertib, respectively. 

LP stability was determined by monitoring their size, PDI and ζ-potential over time. Size and PDI did not undergo significant changes within a month (PDI values remain <0.2 keeping liposomes at room temperature), and the <−20 mV ζ-potential value remained constant for all formulations.

### 3.2. Raman Database of LP Components

**Figure 1:**
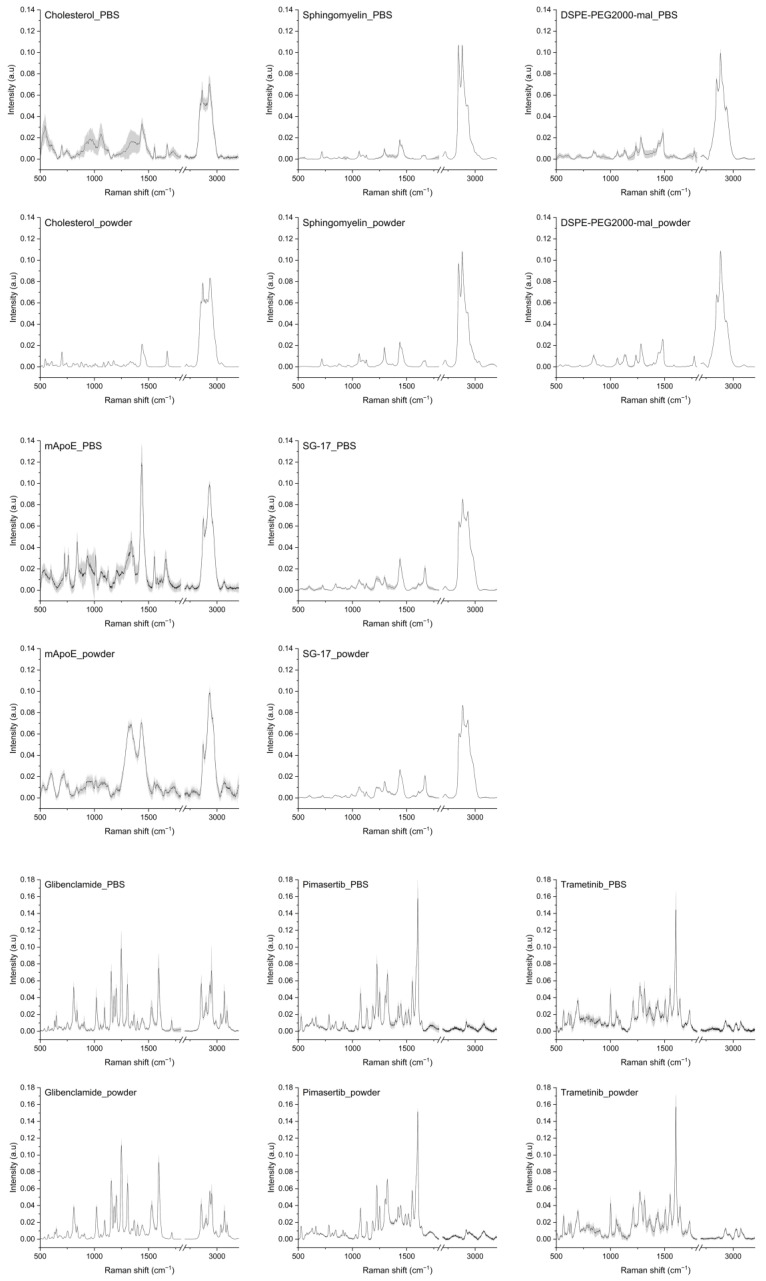
Raman spectra of LP components: cholesterol, sphingomyelin, DSPE-PEG2000-maleimide, mApoE, SG-17, glibenclamide, pimasertib and trametinib in PBS and in solid form (powder). The averages of Raman spectra are represented after baseline subtraction and normalization. Grey areas represent the standard deviations.

In order to study the Raman spectrum of LPs, single components of each nanoformulation were analyzed by collecting their Raman spectra in a database. Raman spectra of Chol, Sm, DSPE-PEG2000-mal, mApoE, SG-17 lipopeptide, glibenclamide, trametinib and pimasertib are represented in Figure 1, both in their solid form and suspended in PBS. 

As for Chol, its spectra as a solid exhibited sharp peaks, while in PBS the peaks widened. In both cases, the main Chol peaks were visible. In particular, peaks at 2867 cm^−1^ and 2933 cm^−1^ were attributed to symmetric stretching of CH_2_ and CH_3_; the peak at 1672 cm^−1^ is related to C=C stretching of the ring. The peak at 1438 cm^−1^ is related to CH_2_ scissoring, and bands at 800–1000 cm^−1^ are related to C-C steroid ring vibration [31]. The spectra of Sm as a powder and in PBS were very similar, with peaks related to C-C stretching (1067 cm^−1^, 1097 cm^−1^, 1130 cm^−1^), CH_2_ deformation (1437 cm^−1^), amide I (1643 cm^−1^), C=C stretching (1673 cm^−1^), O-P-O symmetric stretching (769 cm^−1^) and CN asymmetric and symmetric stretching (957 cm^−1^ and 720 cm^−1^) [32]. Raman spectra of mApoE showed differences when collected on powder and in PBS, but the main prominent peaks were found at 1435 cm^−1^ and in the second spectral region at 2873 and 2930 cm^−1^, related to CH_2_ stretching. As for DSPE-PEG-mal used as a linker for mApoE attachment, its Raman spectrum, in both powder and in PBS, showed peaks at 844, 1280 and 2883 cm^−1^ that can be attributed to the C-O and CH_2_ vibration of DSPE-PEG molecules. Some peaks pertaining to maleimide, in particular at 1063 and 1362 cm^−1^, related to asymmetric and symmetric C-N-C bonds, respectively, and at 1582 cm^−1^ related to C=C stretching [33,34]. Raman spectra of the SG-17 lipopeptide showed peaks in the second spectral region between 2850 and 2930 cm^−1^ for CH_2_ binding, and at 1438 cm^−1^ related to the acyl chain and CH_2_ vibrations.

Regarding drug payloads, their Raman spectra in both forms were very similar. Raman spectra of trametinib, a pyridopyrimidine, showed peaks related to C-C aromatic ring stretching (1002 cm^−1^), C=C in benzenoid ring (1499 cm^−1^) [35] and amide III (1208 cm^−1^). Meanwhile, for pimasertib the most representative peaks were at 1226 cm^−1^ for amide III and at 1422 cm^−1^ for NH binding. Glibenclamide, a second-generation sulfonylurea, presented peaks related to CH_2_ symmetric stretching (1399 cm^−1^), C-C stretching (850 cm^−1^), C=C stretching of the benzene ring (1595 cm^−1^), deformation vibrations of the alkyl groups (1440 cm^−1^), SO_2_ symmetric stretching (1156 cm^−1^) and C-H symmetric stretching (2853 cm^−1^) [36,37]. As stated earlier, the spectra of most components as such and in PBS appeared to be very similar. Since the final LP formulations were in PBS, we decided to consider the spectra of each component in PBS for LP analysis, as they should better represent the LP-encapsulated form. 

### 3.3. Analysis of LP Raman Spectra

**Figure 2:**
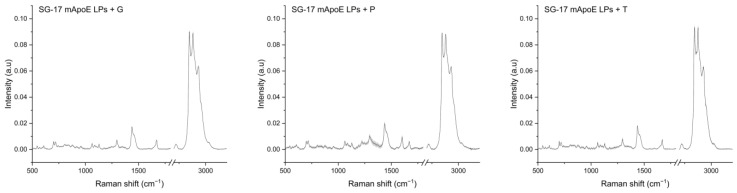
Raman spectra of the SG-17 mApoE LP + glibenclamide/G, SG-17 mApoE LP + pimasertib/P and SG-17 mApoE LP + trametinib/T formulations. Spectra are presented as the average (dark line) after baseline subtraction; grey areas represent the standard deviation.

We analyzed LPs functionalized with mApoE and SG-17 and loaded with glibenclamide, pimasertib or trametinib, as they are conceived as drug-delivery systems for the treatment of AD and GBM. The average spectra of the three formulations are shown in Figure 2. LP Raman spectra seem to be, as expected, a combination of the signals observed with their single components, with prominent peaks confirming their composition.

**Figure 3:**
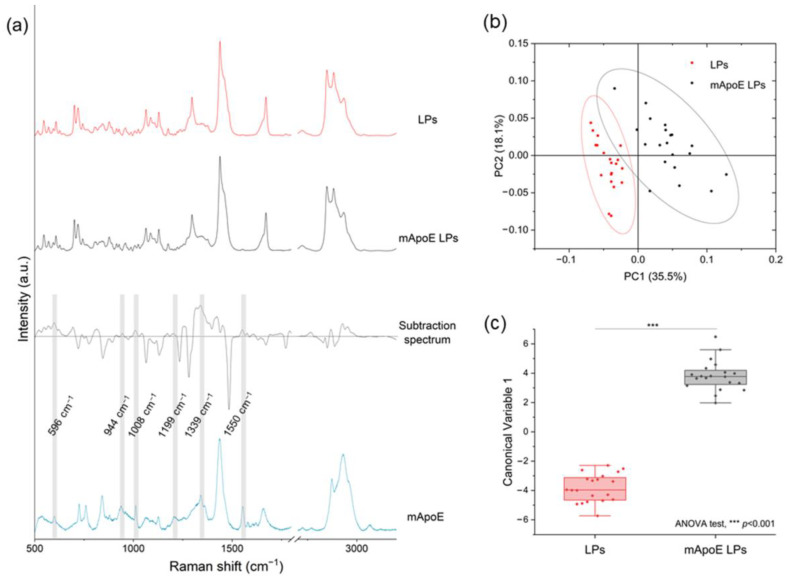
(**a**) LP and mApoE LP spectra, relative subtraction spectrum and mApoE spectrum are represented after baseline subtraction and normalization; (**b**) results of PCA on Raman spectra of LPs and mApoE LPs. Each dot represents one spectrum; (**c**) statistical analysis of the LDA results for the two LPs.

In order to test the ability of RS to identify different LP components, we analyzed and compared the Raman spectra of several liposomal formulations. Firstly, we investigated whether the presence of mApoE on the LP surface influenced the Raman spectrum. Figure 3a shows the average spectra of LPs and mApoE LPs, a subtraction spectrum obtained by subtracting the spectrum of LP from mApoE LP spectrum, and the mApoE spectrum. We observed that some peaks responsible for the difference between the two formulations, shown in the subtraction spectrum, are effectively attributable to mApoE. Indeed, a direct spectral comparison of mApoE with LPs with or without mApoE highlighted slight variations in the signal of the nanocarrier conjugated with the protein. In particular, peaks at 596, 944, 1008, 1199, 1339 and 1550 cm^−1^, highlighted in grey in Figure 3a, are present both in the mApoE spectrum and in the upper area of the subtraction spectrum, which pertains to mApoE LPs. These modifications are prominent enough to determine a significant difference in the PCs distribution obtained after performing PCA on the spectra collected from the two formulations (Figure 3b). Moreover, a PCA-LDA classification made it possible to statistically discriminate the spectra collected from the two formulations, showing that the Canonical Variable 1 scores are significantly different (ANOVA test; Figure 3c, *p* < 0.001).

**Figure 4:**
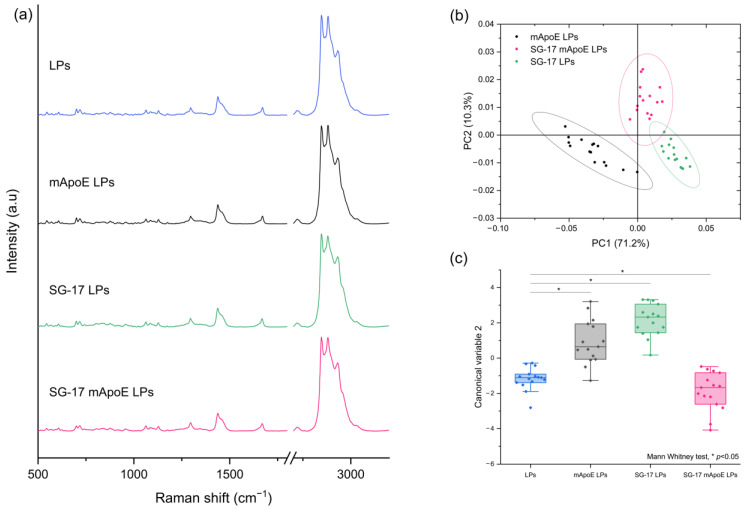
(**a**) Raman spectra of LPs with different surface functionalization: non-functionalized LPs (LPs, blue), LPs functionalized with mApoE (mApoE LPs, black), LPs functionalized with SG-17 (SG-17 LPs, green), LPs functionalized with SG-17 and mApoE (SG-17 mApoE LPs, pink); (**b**) graphical representation of PC1 and PC2 extracted from PCA on mApoE LP, SG-17 LP and SG-17 mapoE LP spectra. The ellipses represent the 95% confidence interval; each dot represents one spectrum; (**c**) statistical analysis (FMann–Whitney test) on Canonical Variable 2 derived from Linear Discriminant analysis for the four formulations.

Secondly, we studied the contribution of the SG-17 lipopeptide in the Raman spectrum. LPs were functionalized with MMP-targeting SG-17 to guarantee a controlled release of the candidate drugs in the diseased environment. For this purpose, we compared the spectra of LPs, mApoE LPs, SG-17 LPs and SG-17 mApoE LPs (Figure 4a). Although these spectra seem to be very similar, the PCA performed on the Raman dataset identified differences among the three functionalized LP formulations. In particular, the presence of SG-17 on the LP surface impacted the LP spectrum, resulting in a distinct separation with mApoE LPs (Figure 4b). 

Furthermore, the Canonical Variables 2 related to the Raman data of the three formulations, obtained by LDA, were statistically different (Mann–Whitney test, *p* < 0.05), demonstrating the ability of RS to differentiate the Raman signal collected from the differently functionalized LP formulations (Figure 4c).

**Figure 5:**
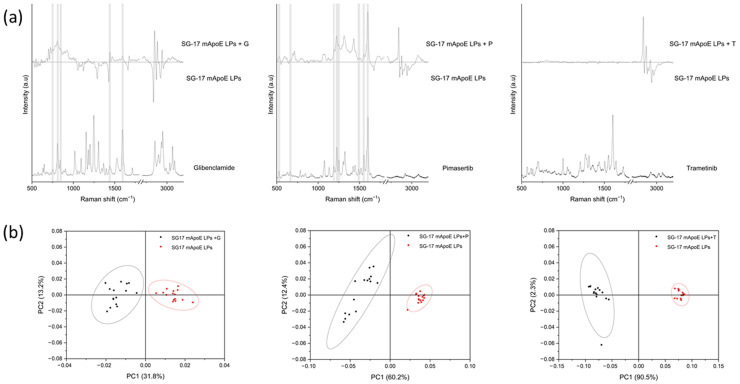
(**a**) Comparison between the subtraction spectrum of three formulations and the spectrum of the relative drugs. The grey bands identify the common peaks; (**b**) results of the PCA performed on SG-17 mApoE LPs loaded (black) and unloaded (red) with three drugs. The ellipses represent the 95% confidence interval; each dot represents one spectrum.

Finally, unloaded SG-17 mApoE LPs and SG-17 mApoE LPs loaded with glibenclamide, pimasertib or trametinib were analyzed. Figure 5a shows the subtraction spectra between corresponding LP formulations with and without the drug, and the Raman spectrum of each drug molecule. Subtraction spectra revealed differences between the Raman fingerprint of the LPs, and the comparison with the spectrum of each pure drug highlighted the presence of common peaks for glibenclamide and pimasertib as payloads (grey bands). Conversely, for LPs loaded with trametinib, spectral modifications were observed only in the 2700–3200 cm^−1^ range, without direct correlations with specific Raman peaks of the drug. However, PCA demonstrated a significant difference between all unloaded and drug-loaded LP pairs, with a widely separated distribution of the first two PCs (Figure 5b). These results suggest that the presence of the drug has an influence on the LP Raman spectrum that can be directly attributable, at least in part, to some predominant, drug-specific peaks (as seen with glibenclamide and pimasertib), or could be related to modifications and/or chemical interactions in the LP structure due to drug loading.

### 3.4. Reproducibility and Stability of Nanoformulations

**Figure 6:**
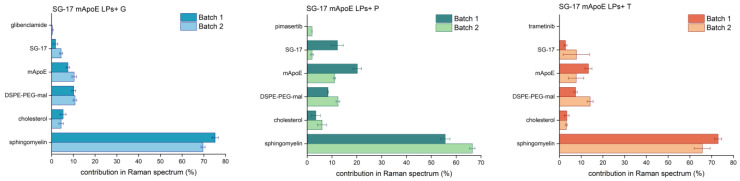
Comparison between different batches of the same formulation. The LP component contribution (%) in the final Raman spectrum was obtained from CLS fitting analysis; average and standard deviation of three spectra for each batch are reported.

CLS fitting was performed on drug-loaded LPs to assess the contribution of single LP components in the spectrum of each formulation. In particular, Raman spectra of multiple batches of the final formulations were acquired to evaluate the reproducibility of the synthesis, but also the suitability of RS as a tool for the quality check of nanoformulations. These analyses (Figure 6) showed a major >50% contribution of Sm in the spectrum of the entire LP. The contribution of Chol, the other constitutive lipid of the LPs, was smaller (lower than 10%). This could be due to Chol being deeply embedded in the phospholipid bilayer, resulting in its masking by Sm molecules. The contribution of other components in the Raman spectrum of LPs was generally around 10–15% for all batches. Interestingly, the Raman spectrum of pimasertib, trametinib and glibenclamide could not be identified in the CLS fitting analysis, suggesting that their signals are hidden by those of other components in the whole Raman spectrum. The average percentage component fit revealed differences between two batches of the same formulations in the Raman fingerprint being constantly less than 14%.

**Figure 7:**
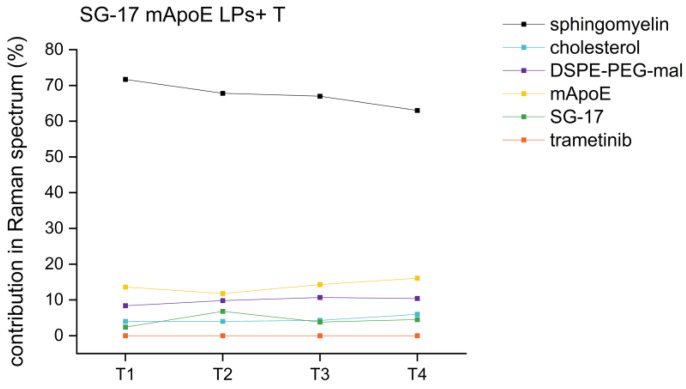
Percentage trends of different LP components in the same formulation (SG-17 mApoE LPs + T) at T1 (one week), T2 (two weeks), T3 (three weeks), T4 (four weeks) after their synthesis.

RS analysis was also exploited for the evaluation of the stability of the nanoformulations. Indeed, the Raman fingerprint of LPs is indicative not only for their biochemical composition, but also for any time-induced degradation. If LPs undergo chemical modifications over time, changes in the Raman results are expected. For this purpose, an SG-17 mApoE LPs + T formulation was analyzed by RS after 1, 2, 3 and 4 weeks from its synthesis, following the same protocol of analysis and the same parameters of Raman spectra acquisition. None of the LP components showed a significant variation in term of percentage scores over time. Indeed, comparing the different time points, the variability is lower than 5% between subsequent weeks (T1-T2, T2-T3 and T3-T4) and lower than 9% within one month from the synthesis, confirming the stability of these LP formulations for at least 1 month (Figure 7).

## 4. Discussion

LPs were synthetized through thin-film hydration followed by probe sonication, and were functionalized for BBB targeting and crossing and for being sensitive to MMPs overexpressed in the inflammatory and tumor microenvironments of brain diseases. These multi-functionalized LPs were loaded with drugs (glibenclamide, pimasertib or trametinib) that need to be released in the disease region to control neuroinflammation and microglial dysfunctions that are impaired in AD and GBM. Before testing their biocompatibility, pharmacokinetics, biodistribution, brain penetrance and the ability to induce therapeutic effects, an accurate characterization of LPs to define their composition, physico-chemical properties and stability is needed, to ensure the reproducibility of their synthesis. 

In the present study, we used RS as a complementary tool to traditional techniques to better comprehend the LP features and the surface modification. Indeed, as a deep characterization of LPs could be a limiting step towards the in vivo efficacy and translation to clinic, the application of RS can provide useful biochemical information through a label-free, low-cost and rapid analysis. At first, we built a Raman database including each single component of the liposomal nanocarriers. The obtained Raman spectra were in line with the spectra reported in the literature for these molecules. For most of them, the Raman spectra as solids were very similar to the spectra in PBS suspensions, with Chol and mApoE showing the largest differences. Moreover, Raman spectra of the full panel of LP formulations were acquired by optimizing the acquisition parameters, in order to record the best signals in terms of intensity and peak definition. For the Raman analysis of these LP-based formulations, Caf_2_ was adopted as a good substrate to deposit a drop of each sample, because it does not interfere with the Raman signals related to the samples. Moreover, the amplification of Raman signals was not required for these samples due to their concentration and chemical compositions. However, in the case of very weak signals, the use of different substrates, such as nanostructured slides covered with metals like gold, would help to enhance the signal intensities through the SERS effect. The obtained spectra showed higher intensities in the 2700–3200 cm^−1^ region, and multiple bands and peaks in the first spectral region (between 500 and 1800 cm^−1^). This trend was similar for all LP formulations, suggesting that the most prominent signal was related to the overall composition of LPs made of Sm, Chol and containing SG-17 and mApoE. Indeed, all spectra have peaks at 1296, 1438, 2850 and 2930 cm^−1^ related to CH_2_ vibrations, and at 1062 cm^−1^ corresponding to C-C stretching, present mainly in the fatty acid chain of Sm and Chol, but also in the peptidic structure of mApoE and SG-17. The mentioned peaks have been identified in other studies aimed at the characterization of different types of LPs by means of RS, to understand their structural deformations induced by factors such as preparation methods, pH, or temperature [38]. 

A deeper analysis of Raman spectra allowed us to investigate the contribution of each component in the spectrum, in order to understand the sensitivity of the analysis to any chemical modifications in the LP composition [39]. Through multivariate analysis, we found that the Raman spectrum of our formulations was more influenced by the presence of SG-17 than of mApoE. Indeed, comparing LPs that differ only for the presence of SG-17, Raman spectra were statistically different, with no overlap in the 95% confidence ellipse of PC1 and PC2 (Figure 4b). Instead, LPs with and without mApoE could still be discriminated, but there was a partial overlap of the 95% ellipses of PC1 and PC2, both when comparing LPs containing SG-17 (Figure 4b), and when comparing LPs without SG-17 (Figure 3b). This result could be due to a more intense signal, or to a larger amount of SG-17 than mApoE attached and exposed on the surface of the LPs to interact with the laser. 

The influence of drug payloads in the LP spectra was investigated by a comparison with drug-loaded LPs, observing a different contribution in the Raman spectrum of LPs according to each drug molecule. In particular, our data suggest that the LP Raman spectra shows specific peaks related directly to glibenclamide and pimasertib, confirming the significant difference with PCA. Conversely, spectra of trametinib-loaded LPs showed differences with respect to unloaded LPs that are not directly related to the trametinib molecule itself. Overall, these data suggest that a drug payload may induce modifications in the molecular interactions within the LP structure that modify the collected Raman signals, thus making it possible to discriminate between formulations according to their composition. 

Together with these data, the CLS fitting analysis revealed some other aspects of the LP characterization through RS. Indeed, CLS fitting makes it possible to evaluate the percentage contribution of each single component in the spectrum of the whole LPs. It is worth noting that the quantification of each component present in the final formulation was beyond the scope of this analysis. We aimed rather to identify a picture of the contribution of each component spectrum in the one of the final LP formulations. This analysis could also identify the main LP components responsible for the potential changes in their Raman spectrum during time and between different batches. For example, CLS fitting has already been proven useful for the analysis of cells to determine cellular components, such as the Raman analysis of human lymphocytes for the identification of biochemical species involved in the development or progression of prostate cancer [40]. In our study, CLS analysis revealed a good level of synthetic reproducibility, showing ≤14% variations in the contribution of each component in the Raman spectrum of different batches of the same LP formulations. Indeed, the study of the reproducibility of LP synthesis is crucial, also because of the method used in this work for the synthesis of the LPs, which could be operator-dependent with respect to other novel methods based on microfluidics [41]. However, our results showed that formulations are reproducible in terms of the presence of the components in the nanostructures revealed by RS. As to the stability of any formulation, the variation of LP components was ≤9% within one month after synthesis, which is in accordance with the analysis of the diameter and PDI values, which were constant for a month; therefore we can assume that the risk of aggregation of these LP is very low. Indeed, it is worth noting that general lipid-based nanoparticles have a risk of aggregation and cluster formation during practical use [42], which has to be considered when developing a novel drug-delivery system. These are encouraging results, considering that both reproducibility and stability assessment are some of the major considerations for LPs production. Size distribution and encapsulation efficiency are important indicators of these properties [43]; however, RS could be a helpful method for evaluating and optimizing the efficacy of LP synthesis. Indeed, despite some intrinsic advantages of RS, to date few studies used this qualitative tool for the investigation of nanoformulations, in particular of LP features. As we noted, RS can be a useful technology for the study of molecule incorporation into LPs, since lipid–molecule interaction leads to changes in the Raman spectrum [44]. Even if not applied to LPs, the ability of RS to study the surface modification and the stability was also confirmed by another work on the analysis of hyaluronic acid-coated and uncoated polymeric nanoparticles [45]. This variety of information obtained by using this technique could pave the way for a greater use of RS to study the physico-chemical characteristics of LPs in support to traditional methods.

## 5. Conclusions

This work represents an innovation in the characterization of LP-based drug-delivery systems that can be extended to different types of nanoparticles for the treatment of many diseases. The formulations presented here were tested for crossing the BBB and releasing specific drugs into the diseased area of the brain; for this reason, they can be potentially developed and optimized for other neurological diseases besides AD and GBM, including the case of hydrocephalus patients, where the BBB has been a great hurdle for drug delivery to the brain [46,47]. Such a deep Raman analysis was proposed here for the first time to study functionalized LPs as therapeutics for brain diseases. The use of small unlabeled sample volumes and a reusable substrate make RS analysis fast and cheap. Obviously, a Raman spectrometer is a bulky and expensive instrument, but replacement with a portable instrument with comparable spectral resolution could overcome these disadvantages. Compared to traditional techniques used for the physico-chemical characterization of substances, RS allowed us to study in a single analysis surface functionalization, drug loading, reproducibility of the synthesis and stability of nanoformulations, using a small amount of sample. In particular, we demonstrated that each component of the LP formulation contributes to the Raman spectrum, making it possible to perform quality control of the preparations by discriminating LPs with different functionalization patterns. With the same fast and simple analysis, it was also possible to verify the reproducibility of the synthesis of the formulations and to demonstrate their stability over time.

However, a limitation of this technology is the impossibility of analyzing fluorescent samples, since fluorescence can totally obscure the Raman effect. 

In conclusion, the obtained results pave the way for the introduction of RS as a quality control tool that requires a small amount of sample and less time with respect to other techniques, with the purpose of supporting and helping the design and synthesis optimization of nanomedicines developed to cure neurological disorders.

## Figures and Tables

**Table 1:** List of different LP formulations that were analyzed using Raman Spectroscopy. x is indicated when the component was included in the formulation, / is indicated when the component is not included in the formulation.

	Cholesterol	Sphingomyelin	DSPE-PEG-Maleimide	mApoE	SG-17	Drug
LPs	x	x	x	/	/	/
mApoE LPs	x	x	x	x	/	/
SG-17 LPs	x	x	x	/	x	/
SG-17 mApoE LPs	x	x	x	x	/	/
SG-17 mApoE LPs + G	x	x	x	x	x	glibenclamide
SG-17 mApoE LPs + T	x	x	x	x	x	trametinib
SG-17 mApoE LPs + P	x	x	x	x	x	pimasertib

## Data Availability

Data sharing not applicable.

## Supplementary Materials

The following supporting information can be downloaded at: https://www.mdpi.com/article/10.3390/nano13040699/s1, Table S1: Raman acquisition parameters of single LP components both in solid form and suspended in PBS; Table S2: Size distribution, diameter and ζ-potential of the multifunctionalized LP formulations; Figure S1: chemical structure of SG-17.
