# Supplementary material for: Raman Spectroscopy Characterization of Multi-Functionalized Liposomes as Drug-Delivery Systems for Neurological Disorders
Source: Nanomaterials (Basel). 2023 Feb 11;13(4):699. doi: 10.3390/nano13040699 (PMC9962107; doi:10.3390/nano13040699)
Supplement: Supplementary file 1 [file nanomaterials-13-00699-s001.zip › nanomaterials-2196589-supplementary.pdf]

Supplementary Material

Raman Spectroscopy Characterization of Multi-Functionalized Liposomes as Drugdelivery Systems for Neurological Disorders

Francesca Rodà [1,2,†], Silvia Picciolini [1†], Valentina Mangolini [1,3], Alice Gualerzi [1], Pierfausto Seneci [4], Antonio Renda [5], SilviaSesana [5], Francesca Re [5] and Marzia Bedoni [1,*]

[1] IRCCS Fondazione Don Carlo Gnocchi ONLUS, Milan, 20148 Italy

[2] Clinical and Experimental Medicine PhD Program, University of Modena and Reggio Emilia, Modena, 41121, Italy

[3.] Department of Molecular and Translational Medicine, University of Brescia, Brescia, 25121, Italy

[4] Chemistry Department, Università degli Studi di Milano, Milan, 20133, Italy

[5.] School of Medicine and Surgery, University of Milano-Bicocca, Vedano al Lambro (MB) 20854, Italy

[†] These authors contribute equally to this work

* Correspondence: Marzia Bedoni; mbedoni@dongnocchi.it; Tel.: +39-02-4030-8874; IRCCS Fondazione Don Carlo Gnocchi, Laboratory of Nanomedicine and Clinical Biophotonics, Via Capecelatro, 66, 20148, Milan, Italy

This supplementary material includes:

- the details of the Raman acquisition parameters used for the analysis of each single component of liposomesto get the most informative Raman fingerprint and create the Raman database (Table S1);
- the information regarding the size distribution, diameter and ζ-potential of the multifunctionalized LPformulations (Table S2);
- the chemical structure of the MMP-sensitive lipopeptide SG-17 (Figure S1).

Table S1. Acquisition parameters used for the Raman analysis of single LP components both in solid form (powder) and suspended in PBS. A few grains of powder and a drop of 3 µl of sample in PBS were lied on a CaF_2 disk and Raman spectra were acquired.

	Laser	Hole	Slit	Grating	Time (s) x accumulation	Delay (s)	Number of spectra
Glibenclamide in PBS	100%	100	400	1800	5x2	10	10
Glibenclamide (powder)	100%	100	400	1800	5x2	12	5
Pimasertib in PBS	100%	100	400	1800	2x2	15	10
Pimasertib (powder)	100%	100	400	1800	2x2	12	5
Trametinib in PBS	100%	100	400	1800	10x2	2	10
Trametinib (powder)	100%	100	400	1800	10x2	2	5
mApoE in PBS	100%	400	400	1800	5x2	5	10
mApoE (powder)	50%	100	400	1800	2x2	5	5
Cholesterol in PBS	100%	400	400	1800	30x2	5	10
Cholesterol (powder)	25%	200	400	1800	2x2	2	5
DSPE-PEG2000-maleimide in PBS	100%	200	400	1800	3x2	8	10
DSPE-PEG-maleimide (powder)	50%	200	400	1800	5x2	5	5
Sphingomielin in PBS	50%	300	400	1800	15x2	5	10
Sphingomielin (powder)	50%	200	400	1800	5x2	5	5
SG-17 in PBS	100%	100	400	1800	4x2	8	10
SG-17 (powder)	100%	100	400	1800	4x2	8	5

Table S2. Size distribution, diameter and ζ-potential of the multifunctionalized LP formulations.

Liposomes	Diameter (nm)	PDI	z-potential (mV)
SG-17 mApoE LPs +G	139±12	0.2	-28±2
SG-17 mApoE LPs + T	169±14	0.2	-37±5
SG-17 mApoE LPs + P	130±8	0.2	-32±3

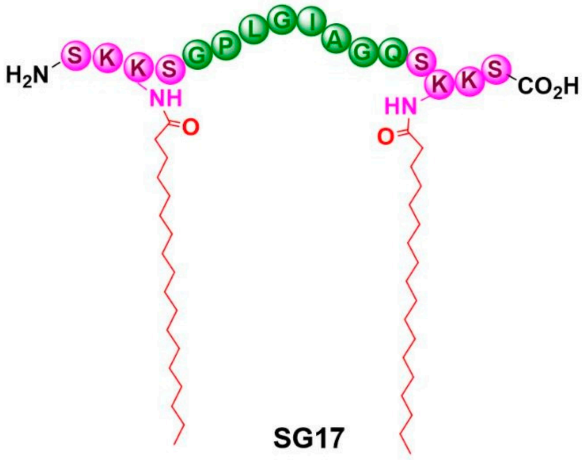

Figure S1. Chemical structure of the lipopeptide SG-17 ((SKK(stearate)SGPLGIAGQSK(stearate)KS), used for targeting specific matrix metallo-proteases (MMPs) overexpressed in the inflammatory and tumor niche.
